# Proton Transfer Charge Reduction Enables Isobaric Labeling-Based Proteoform Quantification of Overlapping Signals in Top-Down Mass Spectrometry

**DOI:** 10.1021/jasms.6c00060

**Published:** 2026-04-22

**Authors:** Philipp T. Kaulich, Andreas Tholey

**Affiliations:** Systematic Proteome Research & Bioanalytics, Institute for Experimental Medicine, Christian-Albrechts-Universität zu Kiel, 24105 Kiel, Germany

**Keywords:** proton transfer charge reduction, top-down proteomics, proteoform, isobaric labeling, quantification

## Abstract

Top-down mass spectrometry provides a powerful approach for analyzing and quantifying intact proteoforms, i.e., the distinct molecular forms of proteins. Isobaric labeling-based quantification strategies offer the advantages of multiplexing and increased analytical depth. However, a major challenge remains the quantification of proteoforms when their precursor signals overlap, leading to mixed reporter ion intensities. In this proof-of-concept study, we employed proton transfer charge reduction (PTCR) at the MS2 level to resolve overlapping precursor signals, allowing selective isolation of individual proteoforms and subsequently, their accurate reporter ion quantification at the MS3 level. Using direct infusion mass spectrometry of model proteins labeled with cysteine-directed tandem mass tags, we demonstrate that this approach enables accurate, interference-free reporter ion-based quantification in the presence of overlapping proteoforms and spectrally congested backgrounds. This work highlights PTCR as a versatile gas-phase separation strategy to enhance the quantitative capabilities of labeling-based top-down mass spectrometry, offering a path toward precise, proteoform-resolved quantification across diverse experimental approaches, such as large-scale top-down proteomics.

## Graphical abstract

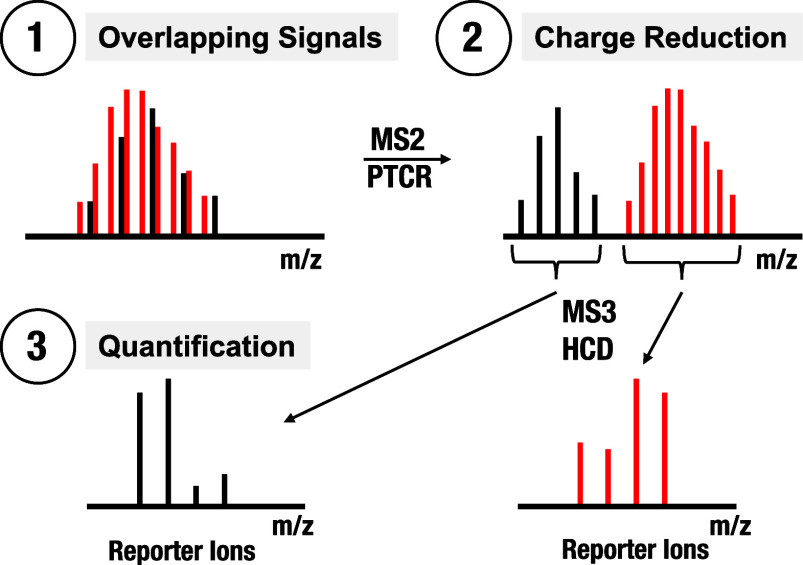

## 1 Introduction

Top-down mass spectrometry (TDMS) is a powerful technique for identifying intact proteoforms, that is, the numerous molecular forms in which proteins can exist.^1^ TDMS is applied across a range of experimental approaches, including single protein characterization, top-down proteomics (TDP), and native TDMS.^2,3^ Besides identification, proteoform quantification is crucial for elucidating molecular processes in biology.^4^ In TDMS, several approaches have been proposed for relative quantification of proteoforms, including label-free quantification (LFQ), stable isotope labeling by amino acids in cell culture (SILAC), and isobaric labeling methods, each with its own advantages and caveats.^5–10^ Isobaric labeling involves the chemical modification of proteoforms with mass-balanced tags that are indistinguishable at the precursor (MS1) level but enable relative quantification through reporter ion detection after fragmentation (MS2). Thus, isobaric labeling approaches enable multiplexed quantification and are readily integrated with multidimensional fractionation strategies; however, they are susceptible to overlabeling and ratio distortion arising from cofragmentation of overlapping proteoforms. Although cysteine-directed reagents such as iodoacetyl tandem mass tags (iodoTMT) reduce overlabeling compared to amino-reactive tags, the challenge of cofragmentation remains a central limitation.^6,7^ For example, we observed a substantial amount of reporter ion signal for identified proteoforms lacking cysteine, which is likely due to cofragmenting proteoforms carrying iodoTMT-labeled cysteines.^11^

Recently, proton transfer charge reduction (PTCR) has been introduced as a valuable tool for proteoform analysis in TDMS.^12–15^ PTCR is a gas-phase ion–ion reaction that reduces the charge states of analyte ions without leading to fragmentation. Charge reduction of precursor ions (i.e., applying PTCR at the MS2 level) facilitates the resolution of highly overlapping signals or large precursor species,^14^ while charge reduction of fragment ions after precursor fragmentation (i.e., PTCR at the MS3 level) enables the separation of overlapping fragment ions, leading to substantially increased sequence coverage.^12^ In addition, PTCR has been shown to support the characterization of proteoform heterogeneity in biotherapeutics.^15^ However, up to now, no quantification workflow with PTCR has been published.

Here, we present a proof-of-concept study using PTCR for labeling-based proteoform quantification in TDMS. By combining MS2 PTCR with MS3 HCD fragmentation, we established a quantitative workflow that enables reporter ion-based quantification in the presence of overlapping proteoform signals and spectrally congested backgrounds. Using direct infusion mass spectrometry for the analysis of iodoTMT-labeled model proteins, we demonstrate that PTCR in combination with MS3 quantification enables quantitation of proteoforms that are inaccessible to conventional MS2-based approaches.

## 2 Methods

### 2.1 Material and Chemicals

Model proteins (bovine serum albumin (BSA), lysozyme, and β-lactoglobulin), chemical reagents, and solvents were purchased from Sigma-Aldrich (St. Louis, Missouri) if not stated otherwise. Cysteine-directed tandem mass tags (iodoTMT, sixplex) were from Thermo Fisher Scientific (San Jose). Deionized water (18.2 MΩ cm^–1^) was prepared by an Arium611 VF system (Sartorius, Göttingen, Germany).

### 2.2 Cys-Directed TMT Labeling

The cysteine-directed iodoTMT sixplex labeling was performed as previously described.^6^ In brief, each of the three model proteins was aliquoted to 2 × 40 μg in 40 μL 6.4 M guanidinium hydrochloride, 200 mM triethylammonium bicarbonate (pH = 8.5). Proteoform reduction was performed by adding 1 μL 100 mM TCEP (37 °C, 800 rpm, 50 min). Each one of the aliquots was labeled with iodoTMT channels 128 and 130, respectively. Only two reporter channels were selected because the objective of this study was to assess the quantitative performance rather than to demonstrate multiplexing capacity. For labeling, the iodoTMT reagents were dissolved in 20 μL of methanol, and 5 μL were added to the samples. Then, the samples were incubated for 50 min at 20 °C and 800 rpm in the dark. Quenching of the reaction was performed by adding 1 μL 100 mM DTT (20 °C, 800 rpm, 50 min). The labeled proteins were combined (channels 128:130; lysozyme 1:1 (v/v), BSA 2:1, β-lactoglobulin 1:2) and purified by methanol-chloroform-water precipitation. Prior to MS analysis, the proteins were resuspended in 50% (v/v) acetonitrile, 1% (v/v) formic acid to a final concentration of ∼0.2 mg/mL.

### 2.3 Direct Infusion MS Analysis

Direct infusion mass spectrometry experiments were performed using an Orbitrap Eclipse Tribrid mass spectrometer (Thermo Fisher Scientific, San Jose) equipped with a heated electrospray ionization source. A flow rate of 3–5 μL/min was delivered using a syringe pump (model F100T2, Chemyx Inc., Stafford, Texas). The typical ion source settings were: spray voltage of +3.5 kV, ion transfer tube temperature of 275 °C, and sheath and auxiliary gas enabled.

The mass spectrometer was operated in “peptide mode” (i.e., the gas pressure in the ion-routing multipole (IRM) was 0.008 Torr). Note that lower IRM pressures (i.e., as utilized in the “protein mode”) result in the loss of reporter ions.^6^ Typical MS settings were: for MS1 scans 120,000 resolution, 246 ms maximum injection time, 100% AGC target, and 2 microscans (except for the BSA-lactalbumin sample, for which the data were acquired using a resolution of 7,500 and 8 microscans); for MS2 PTCR scans, 1.6–5 *m*/*z* isolation window, 5 ms PTCR reaction time (200,000 reagent target, 200 ms maximum reagent time), 120,000 resolution, 246 ms maximum injection time, 1000% AGC target, 1–8 microscans; for the MS2 and MS3 HCD scans, 1.6.-5 *m*/*z* isolation window, 80 V (absolute) collision energy, 30,000 resolution, 54 ms maximum injection time, 1000% AGC target, and 1–8 microscans. A PTCR reaction time of 5 ms was used to achieve sufficient charge reduction while avoiding excessive signal dispersion across many charge states, which would reduce MS3 sensitivity. Wider isolation windows were applied for MS3 approaches due to the reduced spectral complexity of the charge-reduced ion populations and to improve sensitivity. A collision energy of 80 V was applied to ensure efficient reporter ion release, as MS2 and MS3 HCD spectra were used exclusively for quantification.^6^ The acquired mass spectra were analyzed using FreeStyle (v1.8 SP1, Thermo Fisher). Monoisotopic masses were determined by FLASHDeconv^16^ within FLASHApp.^17^

## 3 Results and Discussion

### 3.1 PTCR Preserves Reporter Ion Signal Intensities

To assess whether PTCR affects reporter ion quantification, lysozyme aliquots labeled with iodoTMT channels 128 and 130, respectively, were mixed at an equimolar ratio and analyzed by direct infusion mass spectrometry. The MS1 spectrum exhibited a series of well-resolved signals that could be assigned to charge states ranging from +10 to +26 (Figure 1a). Deconvolution resulted in a monoisotopic mass of 16,937.56 Da, which is in agreement with the theoretical mass expected for lysozyme containing eight iodoTMT-labeled cysteine residues (Δ*M* = −7.68 ppm).

**Figure 1:**
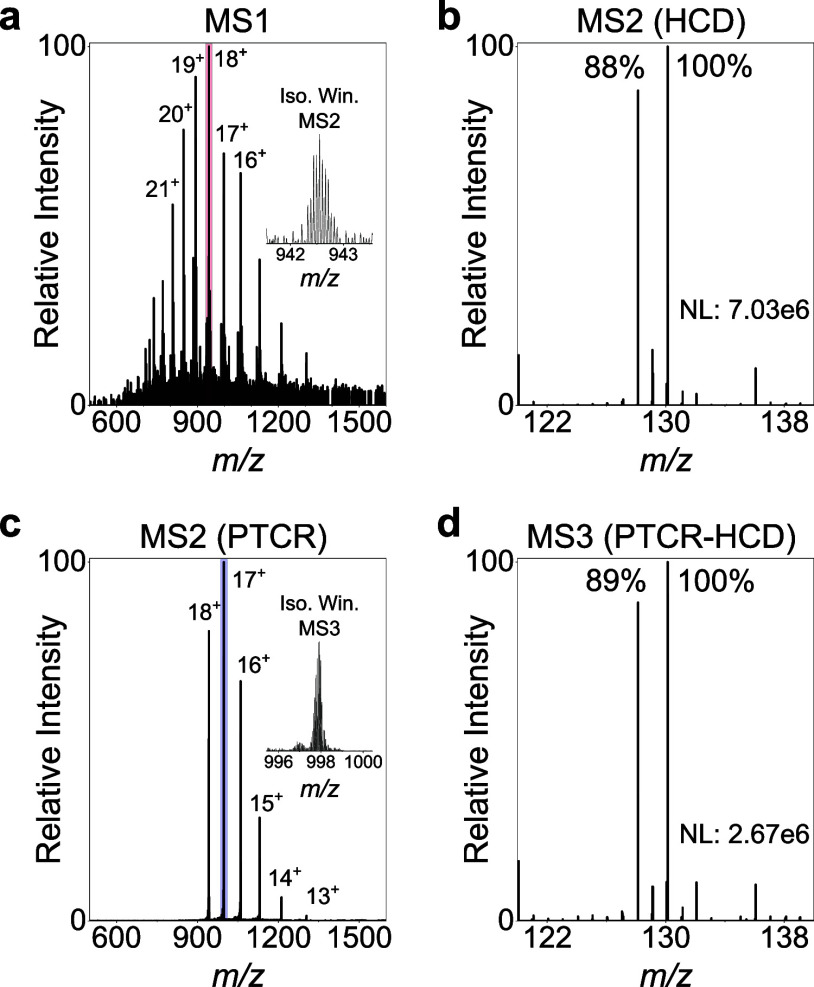
PTCR preserves reporter ion signal intensities. Labeled lysozyme aliquots (iodoTMT, channels 128 and 130) were mixed in a 1:1 (v/v) ratio and analyzed by direct infusion mass spectrometry. (a) Summed MS1 spectrum (insert and red box: isolation window utilized for MS2 acquisition). (b) MS2 HCD quantification spectrum of the +18 charge state. (c) MS2 PTCR spectrum of the +18 charge state, resulting in charge-reduced species (insert and blue box: isolation window utilized for MS3 acquisition). (d) MS3 HCD quantification spectrum of the charge-reduced +17 precursor of the MS2 PTCR scan. Additionally, a diagnostic ion for unmodified lysine residues was detected at *m*/*z* 129.1022,^18^ as well as immonium ions of phenylalanine (*m*/*z* 120.0813) and tyrosine (*m*/*z* 136.0762). Note that at a resolution of 30k, these ions do not interfere with the sixplex quantification. NL, normalization level.

An MS2 HCD scan (80 V) of the +18 charge state yielded TMT reporter ion signals with a ratio close to the expected 1:1 (128:130), with the slight deviation most likely attributable to pipetting errors during channel mixing (Figure 1b). The same ratio was consistently observed across different charge states (Figure S1). Following this, PTCR was applied at the MS2 level (5 ms reaction time), resulting in charge reduction of the precursor to charge states as low as +11 (Figure 1c). Subsequently, the +17 charge-reduced precursor was selected and fragmented by HCD fragmentation (80 V) at the MS3 level. This produced reporter ion signals with the same quantitative ratio as observed at the MS2 level (Figures 1d, S1), demonstrating that PTCR effectively enables charge manipulation without compromising reporter ion-based quantification. The reporter ion intensity (normalization level, NL) at the MS3 level was approximately 40% of that at the MS2 level. The lower intensity in the MS3 workflow is expected, as PTCR distributes the signal across multiple charge states, of which only one is selected for MS3, and additional ion losses occur during ion isolation and transfer.

### 3.2 PTCR Enables Accurate Proteoform Quantification of Overlapping Signals

Next, we investigated whether PTCR enables the separation of overlapping proteoforms and accurate quantification using the MS2 (PTCR)-MS3 (HCD) approach. For this, iodoTMT-labeled lysozyme (128:130 = 1:1) and β-lactoglobulin (128:130 = 1:2) were mixed (1:1, v/v) and analyzed (Figure 2). The MS1 spectrum revealed two clearly distinguishable charge-state series, which could be assigned to β-lactoglobulin and lysozyme, respectively. The charge state distribution corresponding to β-lactoglobulin ranged from +15 to +26. The deconvolved monoisotopic mass was 19,915.54 Da, consistent with β-lactoglobulin including five iodoTMT-labeled cysteine residues (Δ*M* = −0.56 ppm). The charge state distribution and monoisotopic mass of lysozyme were consistent with those described above.

**Figure 2:**
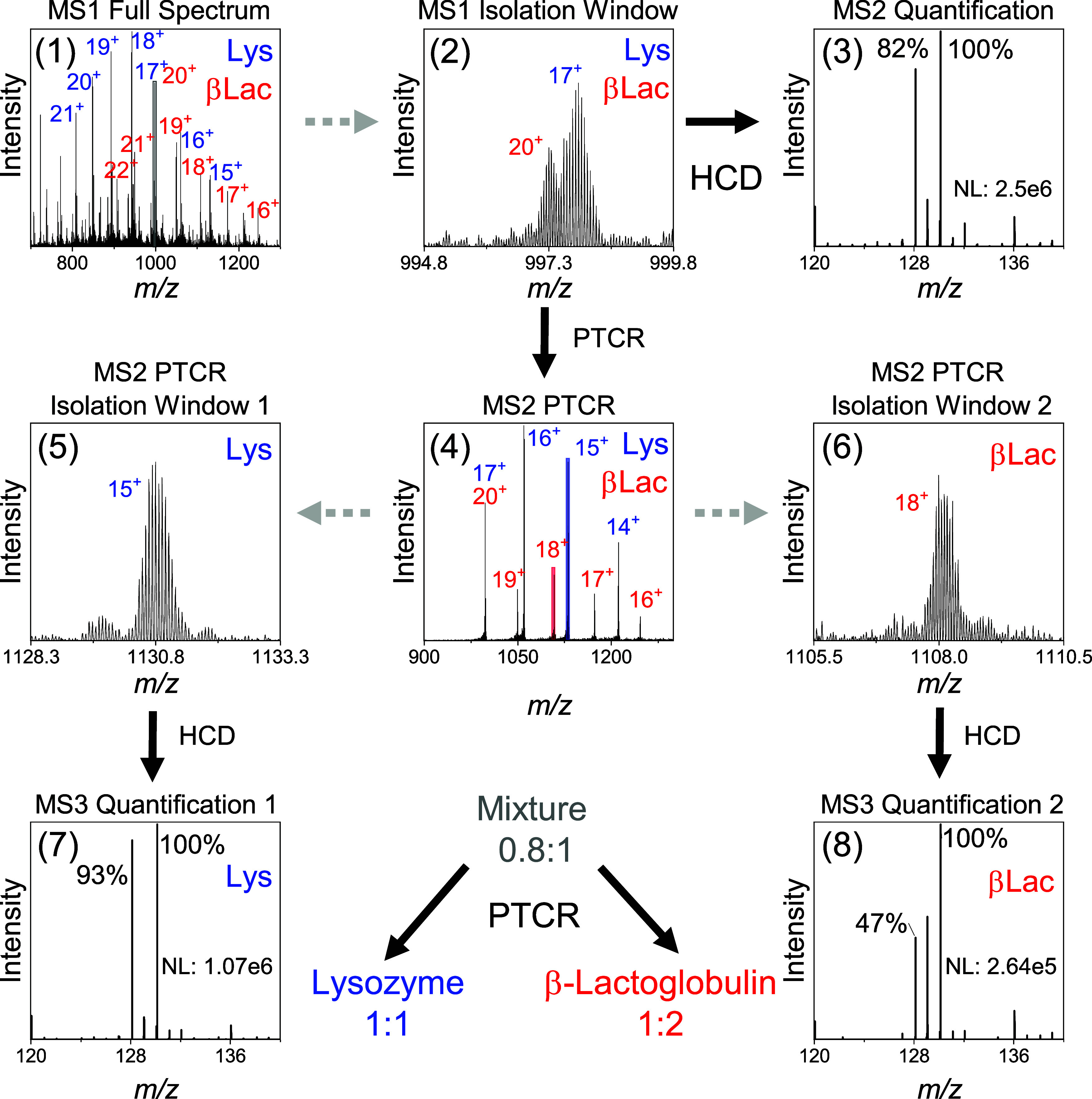
PTCR enables accurate quantification of overlapping proteoforms. Aliquots of labeled (iodoTMT, channels 128 and 130) lysozyme (ratio 1:1) and β-lactoglobulin (ratio 1:2) were mixed (1:1, v/v) and analyzed by direct infusion mass spectrometry. First, a (**1**) summed MS1 spectrum was acquired, showing the charge envelopes of lysozyme and β-lactoglobulin (gray box: MS2 isolation window). (**2**) Selecting overlapping signals for (**3**) MS2 HCD fragmentation resulted in distorted TMT ratios. (**4**) MS2 PTCR produced charge-reduced, nonoverlapping lysozyme and β-lactoglobulin species. From the charged-reduced species, (**5**, **6**) certain precursors were selected (red and blue boxes: MS3 isolation windows) and subjected to (**7, 8**) MS3 HCD fragmentation, resulting in accurate quantification of (**7**) lysozyme and (**8**) β-lactoglobulin.

Since the +17 charge state of lysozyme overlapped with the +20 charge state of β-lactoglobulin (∼996.5–998.5 *m*/*z*), this precursor population was selected to serve as a model system for overlapping proteoforms in this proof-of-concept study (Figure 2). As expected, an MS2 HCD quantification scan yielded a reporter ion ratio of 0.8:1, reflecting mixed contributions from both proteoforms due to cofragmentation. Applying MS2 PTCR to this overlapping precursor population results in efficient charge reduction for both proteoforms: Lysozyme exhibited charge states ranging from +12 to +17, whereas β-lactoglobulin exhibited charge states from +15 to +20, with no overlap among the charge-reduced species of the two proteoforms.

Following MS2 PTCR, charge-reduced species corresponding to lysozyme (charge state +15) and β-lactoglobulin (+18) could be selectively isolated. Subsequent MS3 HCD fragmentation yielded reporter ion signals that reflected the original labeling ratios of the respective proteoforms. Specifically, lysozyme exhibited a reporter ion ratio of 1:1, whereas β-lactoglobulin showed a ratio of 1:2 (Figure 2).

These results demonstrate that PTCR resolves overlapping proteoforms that cannot be quantified at the MS2 level. By providing an additional gas-phase dimension for precursor separation, PTCR prevents distortion of reporter ion ratios by enabling the selection of nonoverlapping precursors for quantification at the MS3 level.

### 3.3 Proteoform Quantification in a Spectrally Congested Background

Furthermore, we evaluated proteoform quantification in the presence of a spectrally congested background. For this purpose, iodoTMT-labeled bovine serum albumin (BSA; 128:130 = 2:1) and β-lactoglobulin (ratio 128:130 = 1:2) were mixed (1:1, v/v) and analyzed by direct infusion mass spectrometry. The resulting MS1 spectrum lacked discernible charge-state distributions but instead consisted of a dense collection of signals (Figure 3a). This observation is characteristic of denatured TDMS for large, heterogeneous proteins such as BSA, which comprises a plethora of proteoforms,^19^ resulting in many overlapping, indistinguishable signals (Figure S2).

**Figure 3:**
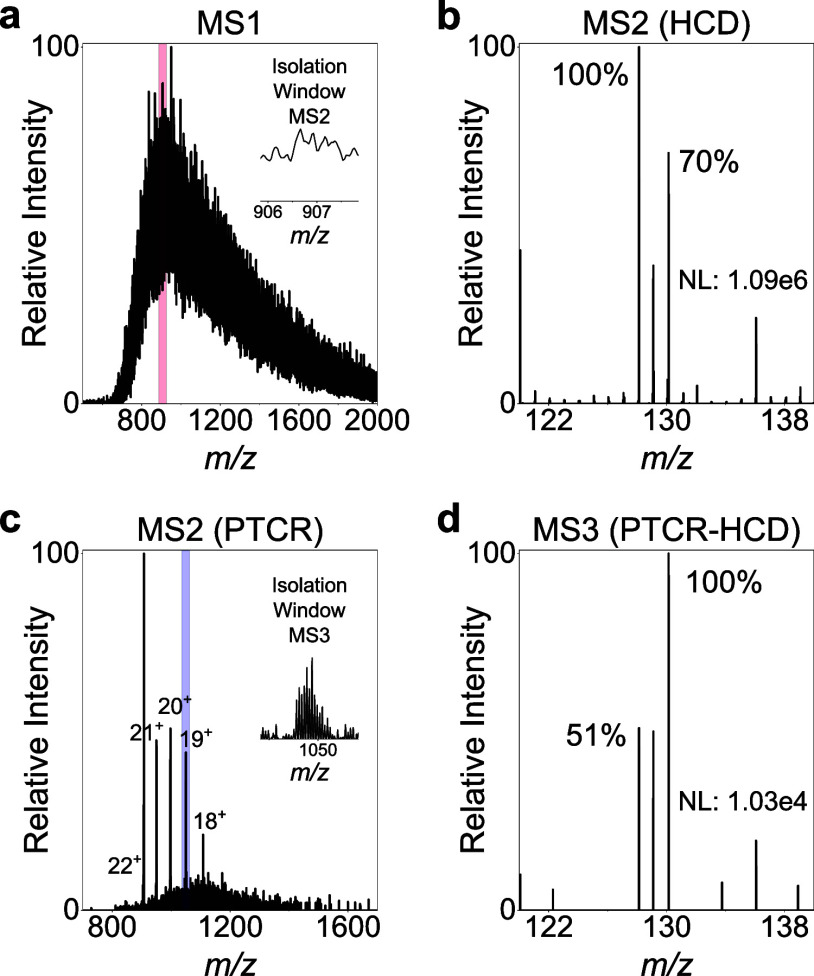
PTCR enables accurate quantification of proteoforms in spectrally congested backgrounds. Aliquots of labeled (iodoTMT, channels 128 and 130) β-lactoglobulin (ratio 1:2) and BSA (ratio 2:1) were mixed (1:1, v/v) and analyzed by direct infusion mass spectrometry. (a) Summed low-resolution MS1 spectrum (insert and red box: isolation window for MS2). (b) MS2 HCD quantification spectrum of the +22 charge state of b-lactoglobulin. (c) MS2 PTCR spectrum of the +22 charge state of β-lactoglobulin (insert: isolation window for MS3). (d) MS3 HCD quantification spectrum of the charge-reduced +19 precursor after MS2 PTCR.

Under these conditions, targeted MS2-based reporter ion quantification was performed by selecting the *m*/*z* region in which the charge state +22 of β-lactoglobulin was expected (even though no distinct β-lactoglobulin charge state was observable). Fragmentation of this precursor population generated reporter ion signals with a ratio of 1:0.7 (Figure 3b). Due to the high spectral complexity within the selected *m*/*z* range, the measured ratio reflected contributions from multiple cofragmented species (i.e., BSA proteoforms and β-lactoglobulin), hampering unambiguous, proteoform-specific quantification.

Therefore, an MS2 PTCR experiment was performed, in which the selected precursor population was subjected to charge reduction. The resulting PTCR spectrum revealed a series of well-defined charge states from β-lactoglobulin (+17 to +22), although low-intensity, unresolved background signals from coisolated BSA species remained (Figure 3c). Isolation of the +19 charge-reduced β-lactoglobulin precursor followed by MS3 HCD fragmentation yielded a reporter ion ratio consistent with the expected labeling ratio (1:2), indicating minimal interference from coisolated species (Figure 3d). These results demonstrate that PTCR enables accurate reporter ion-based quantification of proteoforms even in the presence of a highly dense, spectrally congested background.

## 4 Conclusion

In this proof-of-concept study, we addressed the challenge of overlapping proteoform signals, which is a key limitation of labeling-based TDMS quantification approaches. Co-fragmentation of multiple proteoforms leads to mixed reporter ion intensities and hinders accurate proteoform quantification. Applying PTCR resolved overlapping precursors by generating charge-reduced species. Subsequent isolation of nonoverlapping signals and HCD fragmentation at the MS3 level enabled accurate reporter ion-based quantification of individual proteoforms. Furthermore, we demonstrated that this approach can also be used for highly crowded spectra (e.g., in the presence of substantial background signal), enabling selective isolation and accurate reporter ion-based quantification of individual proteoforms. While cysteine-directed labeling (iodoTMT) was applied in this work,^6^ the approach is compatible with other isobaric labeling strategies, such as amino-reactive TMT.^7^

In isobaric labeling-based TDMS, quantification is typically performed using a two-scan strategy comprising separate MS2 identification and quantification scans of the same precursor, allowing the use of optimized fragmentation conditions for each purpose.^11^ Extension of this concept to the MS3 level may enable improved identification of coisolated species. Alternatively, proteoform identification can be achieved by analyzing chimeric MS2 spectra^20^ in combination with intact proteoform mass information derived from MS2 PTCR.^13^

For implementing the MS3-based quantification approach in LC- or CE-MS workflows, the additional acquisition time required for MS2 PTCR and MS3 HCD, as well as the reduced sensitivity of MS3 reporter ion generation, must be considered, as they may limit overall throughput. However, PTCR-based acquisition strategies have already been successfully demonstrated on chromatographic time scales.^13^ Future developments in intelligent data acquisition,^21^ such as the selective application of PTCR to coisolated precursor populations, may further improve the efficiency and applicability of our approach.

In summary, this study demonstrates a practical strategy for addressing overlapping proteoform signals in reporter ion-based proteoform quantification in TDMS. By resolving overlapping precursors in the gas-phase, this approach is expected to be applicable to LC- or CE-MS-based TDP workflows, where spectral congestion and coisolation remain persistent challenges. Furthermore, this work demonstrates that labeling-based quantification enables quantitative analysis in PTCR-based workflows.

## Supplementary Material



## Data Availability

The acquired spectra have been uploaded to the ProteomeXchange Consortium via the PRIDE partner repository with the data set identifier PXD074730.^22^

## Notes

Reproducibility of reporter ion quantification across various charge states (Figure S1); high-resolution mass spectrum of BSA (Figure S2) (PDF)
