# Supplementary material for: Proton Transfer Charge Reduction Enables Isobaric Labeling-Based Proteoform Quantification of Overlapping Signals in Top-Down Mass Spectrometry
Source: J Am Soc Mass Spectrom. 2026 Apr 22;37(5):1289–94. doi: 10.1021/jasms.6c00060 (PMC13154202; doi:10.1021/jasms.6c00060)
Supplement: Supplementary file 1 [file js6c00060_si_001.pdf]

Supporting Information

Proton Transfer Charge Reduction Enables Isobaric Labeling-Based Proteoform Quantification of Overlapping Signals in Top-Down Mass Spectrometry

Philipp T. Kaulich*, Andreas Tholey

Systematic Proteome Research & Bioanalytics, Institute for Experimental Medicine, Christian-Albrechts-Universität zu Kiel, 24105 Kiel, Germany

* to whom correspondence should be addressed:

Philipp T. Kaulich

Systematic Proteome Research & Bioanalytics, Institute for Experimental Medicine

Christian-Albrechts-Universität zu Kiel

24105 Kiel, Germany

Phone: #49 (431) 50030355; Fax: #49 (431) 50030308

E-mail: p.kaulich@iem.uni-kiel.de

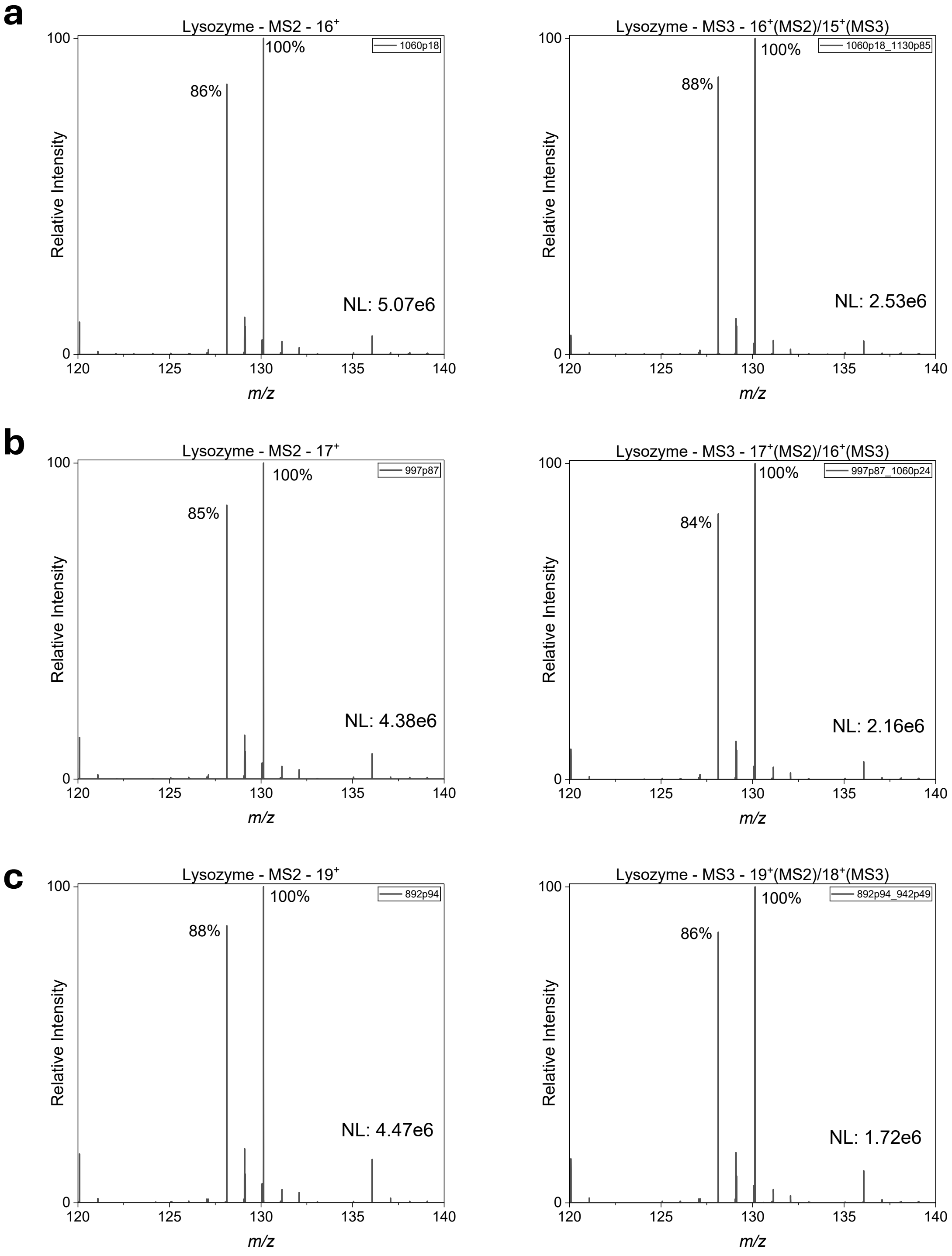

Supplementary Figure S1: **Reproducibility of reporter ion quantification across various charge states**. Labeled lysozyme aliquots (iodoTMT, channels 128 and 130) were mixed in a 1:1 (v/v) ratio and analyzed by direct infusion mass spectrometry. **a-c** Different charge states were isolated (**a** 16^+, **b** 17^+, **c** 19^+) and either subjected to MS2 HCD fragmentation (left panels) or to MS2 PTCR followed by isolation of charge states increased by one and subsequent MS3 HCD fragmentation (right panels).

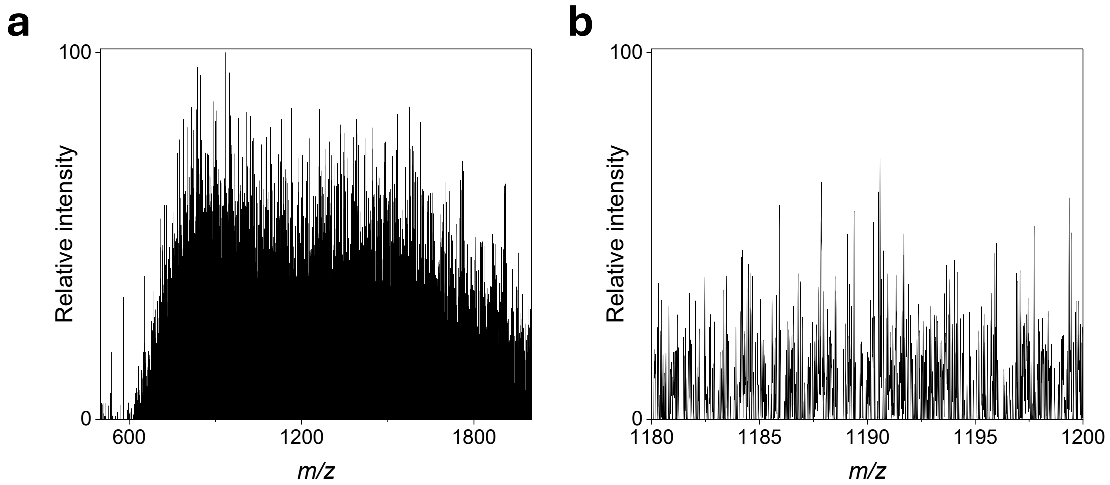

Supplementary Figure S2: **High-resolution mass spectrum of BSA**. Aliquots of labeled (iodoTMT, channels 128 and 130) BSA (ratio 2:1) were mixed (1:1, v/v) and analyzed by direct infusion mass spectrometry. **a** High-resolution full MS spectrum. **b** Zoom to *m/z* 1180-1200 in the high-resolution MS1 spectrum.
